# Pericentriolar Targeting of the Mouse Mammary Tumor Virus GAG Protein

**DOI:** 10.1371/journal.pone.0131515

**Published:** 2015-06-29

**Authors:** Guangzhi Zhang, David Sharon, Juan Jovel, Lei Liu, Eytan Wine, Nasser Tahbaz, Stanislav Indik, Andrew Mason

**Affiliations:** 1 Division of Gastroenterology, Department of Medicine, University of Alberta, Edmonton, Alberta, Canada; 2 Department of Pediatrics, University of Alberta, Edmonton, Alberta, Canada; 3 Department of Cell Biology, University of Alberta, Edmonton, Alberta, Canada; 4 Research Institute for Virology and Biomedicine, University of Veterinary Medicine Vienna, Vienna, A-1210, Austria; University of Alabama at Birmingham, UNITED STATES

## Abstract

The Gag protein of the mouse mammary tumor virus (MMTV) is the chief determinant of subcellular targeting. Electron microscopy studies show that MMTV Gag forms capsids within the cytoplasm and assembles as immature particles with MMTV RNA and the Y box binding protein-1, required for centrosome maturation. Other betaretroviruses, such as Mason-Pfizer monkey retrovirus (M-PMV), assemble adjacent to the pericentriolar region because of a cytoplasmic targeting and retention signal in the Matrix protein. Previous studies suggest that the MMTV Matrix protein may also harbor a similar cytoplasmic targeting and retention signal. Herein, we show that a substantial fraction of MMTV Gag localizes to the pericentriolar region. This was observed in HEK293T, HeLa human cell lines and the mouse derived NMuMG mammary gland cells. Moreover, MMTV capsids were observed adjacent to centrioles when expressed from plasmids encoding either MMTV Gag alone, Gag-Pro-Pol or full-length virus. We found that the cytoplasmic targeting and retention signal in the MMTV Matrix protein was sufficient for pericentriolar targeting, whereas mutation of the glutamine to alanine at position 56 (D56/A) resulted in plasma membrane localization, similar to previous observations from mutational studies of M-PMV Gag. Furthermore, transmission electron microscopy studies showed that MMTV capsids accumulate around centrioles suggesting that, similar to M-PMV, the pericentriolar region may be a site for MMTV assembly. Together, the data imply that MMTV Gag targets the pericentriolar region as a result of the MMTV cytoplasmic targeting and retention signal, possibly aided by the Y box protein-1 required for the assembly of centrosomal microtubules.

## Introduction

The Gag protein plays a pivotal role in dictating the subcellular localization of immature capsid assembly [1–3]. For example, betaretroviruses assemble immature capsids in the cytoplasm while alpharetroviruses, gammaretroviruses and lentiviruses assemble at the inner plasma membrane. HIV Gag is the only structural protein required for particle formation and plasma membrane localization [4, 5], mediated by a bipartite signal located in the matrix (MA) domain, which involves both a N-terminus myristoylation signal and a stretch of basic residues [6–10]. Even though betaretroviruses also have a myristoylation signal, the immature capsids assemble intra-cellularly as a result of a cytoplasmic targeting/retention signal (CTRS) in the MA domain [1, 2, 11]. This site was first discovered in the Mason–Pfizer monkey virus (M-PMV) using mutational analyses resulting redistribution of viral assembly from the cytoplasm to the plasma membrane [1, 3]. Subsequently, the Gag polyproteins of Jaagsiekte sheep retrovirus (JSRV) and foamy virus (FV) were found to assemble as capsids at the pericentriolar region [12–16], suggesting that this might be a conserved site for retroviral assembly.

Mouse mammary tumor virus (MMTV) is a complex retrovirus encoding structural (Gag, Env), replication-associated (Pro, Pol, Dut) and regulatory proteins (Sag, Rem) [17]. The MMTV Gag polyprotein is translated from full-length unspliced genomic RNA and requires the regulatory protein Rem for efficient translation [18–20]. Gag is assembled in the cytoplasm prior to transport to the plasma membrane for budding, where the polyprotein is processed by the viral protease into its constituent mature proteins NH2-MA, pp21, p3, p8, n, CA, NC-COOH [21].

The MA domain of the MMTV Gag contains an N-terminus myristoylation site, which is considered essential for plasma membrane trafficking as deletion abolishes virus budding [22]. The MMTV p3-p8-n domains are likely involved in morphogenesis as deletion results in the prototypic spherical form changing to a rod-shaped virion [23]. The p3-p8 domain is homologous to the p12 of M-PMV Gag, which contains an internal scaffold domain responsible for promoting Gag self-interaction [24]. Whilst self-interaction of MMTV p3-p8 remains to be demonstrated, the homology between p3-p8 and p12 suggests that MMTV Gag oligomerization may require the concerted action of its multiple domains in addition to the NC region [24].

The study of MMTV Gag assembly has been limited to date. A recent report proposes that Gag co-localizes with the ribosomal protein L9 in a subset of MMTV-infected cells suggesting that nucleolar localization maybe required for virion assembly [25]. In the cytoplasm, MMTV Gag co-localizes with viral RNA and YB-1, a translational regulator associated with P bodies and stress granules [26]. Notably, YB-1 plays an important role in centriolar and centrosome maturation [27] and its knockdown results in diminished MMTV particle production [26]. Therefore, MMTV assembly appears to be a multi-step process involving the interaction of host and Gag proteins in several subcellular compartments.

As other retrovirus Gag proteins assemble adjacent to the centriole [13, 14, 16], we tested the hypothesis that MMTV Gag may assemble in a similar location because MMTV contains a CTRS site in the MA domain [1, 11]. We conducted studies on MMTV assembly in both mouse and human cell lines because recent reports have documented that MMTV GR and C3H can form productive infection in human cells [28] and MMTV-like proviral integration sites from the closely related human betaretrovirus (HBRV) can be readily detected in patients with autoimmune liver disease [29]. We synthesized an MMTV Gag-GFP fusion construct by inserting the MMTV 5’ untranslated region (UTR) upstream of the gag open reading frame (ORF) to optimize protein expression. During both transient Gag-GFP expression and viral infection, we observed that MMTV Gag localized to the pericentriolar region by confocal microscopy and transmission electron microscopy (TEM). We also found that the MMTV MA domain was involved with cytoplasmic localization around the pericentriolar region, as described for other retroviruses.

## Materials and Methods

### Recombinant DNA expression constructs

All MMTV sequences were derived from pGR102, a molecular clone of MMTV [30]. To generate p-Gag-EGFP expression plasmid, in which the GFP was cloned at the C-terminus of the Gag protein, the egfp (Clontech) and the gag open reading frames were PCR-amplified with primer pairs 1/2 and 3/4, respectively (Table 1), using HiFi Taq polymerase (Invitrogen). The amplified fragments were digested and inserted at the *Kpn*I/*Bam*HI and *Nhe*I/*Kpn*I sites of pcDNA3.1 (Invitrogen), respectively. Construction of p-UTR-Gag-GFP and p-MA-GFP was performed in a similar manner using the primer sets 5/4, 8/5, respectively, and the amplified fragments were digested and inserted into the *Nhe*I and *Kpn*I sites of pGag-GFP. To generate pGag-GFP-CTE, CTE was amplified from plasmid p-M-PMV CTE (provided by Dr. Tahir Rizvi, United Arab Emirates University, UAE) with primer set 6/7 and the amplified fragment was digested and inserted into the *Xba*I site of plasmid pGag-GFP. The p-CMV-MMTV, where the full length MMTV was placed under the control of the CMV promoter, has been described previously [31]. To construct p-Gag, the *Hind*III and *Kpn*I fragment from p-CMV-MMTV was excised and inserted into the corresponding sites of p-Gag-GFP-CTE. To construct p-Gag-Pol, the sequence encoding the Pro-Pol was amplified with primer pair 11/12 and inserted into the *Kpn*I and *Xho*I sites of p-Gag. To construct p-CMV-MMTV-puromycin, the puromycin open reading frame was amplified with primer pair 9/10 and inserted into the *Sma*l/*Bst*BI sites of p-CMV-MMTV. Homologous recombination [32] was used to generate p-Gag-GFP_191_ and p-Gag-GFP_D56/A_. To construct p-Gag-GFP_191_, the p-EGFP and p-Gag were amplified with primer pairs 13/14 and 15/16, respectively. The PCR products were mixed at a 1:1 ratio, digested with *DpnI* for 1 hour at 37°C to cleave template plasmid and then transformed into DH5α cells. The p-Gag-GFP_191_ plasmid positive colonies were screened from the transformed bacteria. The p-Gag-GFP_D56/A_ was constructed in a similar manner using primer 17/18 and p-Gag-GFP_191_ as template. All primers used for cloning are listed in Table 1.

**Table 1:** Primer sequences for constructing expressing plasmids and qPCR.

No.	Primer sequence (5’ to 3’) ^a^	Clones (restriction enzyme
1	GTTGGGTACCGGAGGCGGTAGTATGGTGAGCAAGGGCG	5’ EGFP-GGGS (KpnI)
2	TCGAGGATCCTTACTTGTACAGCTCGTCCA	3’ EGFP (BmaHI)
3	ATCGGCTAGC ATGGGGGTCTCGGG	5’ p-Gag-GFP (NheI)
4	CTCCGGTACCCAAGTTTTTTGAATTTTC	3’ p-GagGFP (KpnI)
5	ATTCGCATGCGCAACAGTCCTAACATTCACCTC	5’ p-UTR-Gag-GFP (NheI)
6	AAAATCTAGAACCTCCCCTGTGAGCTAGACTG	5’ p-CTE (Xbal)
7	AAAATCTAGAACACA TCCCTCGGAG GCTGC	3’ p-CTE (Xbal)
8	TCCGGTACCTAGCAAAACCAAGTCTGATTGC	3’ p-MA-GFP (KpnI)
9	GATCGATATCCCGGGATGGCCACCGAGTACAAGCCCAC	5’ p-CMV-MMTV-purimycin (Smal)
10	GATCTTCGAATCAGGCACCGGGCTTGCGGGTC	3’ p-CMV-MMTV-purimycin (BstB1)
11	TAAAGGTACCCTCCCTGAAGGGACCA	5’ p-Gag-Pol (KpnI)
12	TAGTCCTCGAGTTAAGGACCTCCTCCG	3’ p-Gag-Pol (XhoI)
13	GTAAGAGAAAAGAGAAGGGCGGCATGGTGAGCAAGGGCG	GFP insertion 190KD FW
14	TAAAAAGGCCTTCTGATCCTTGTACAGCTCGTCCA	GFP insertion 190KD RV
15	GATCAGAAGGCCTTTTTAGCCAC	102 Gag insertion 190KD FW
16	CTTACCCTCACCAATTTCTTTTCTTTG	102 Gag insertion 190KD RV
17	TCACAAGCTTGGAAGAGGGTAGGAAGAGAATG	54 D/A FW
18	CTCTTCCAAGCTTGTGAATTTAATCCTCCTTCTTCG	54 D/A RV
19	GACCACTACCAGCAGAACAC	p-Gag-GFP forward
20	GAACTCCAGCAGGACGATG	p-Gag-GFP reverse
21	TGACAGGATCGAGAAGGAGA	Human β-actin forward
22	CGCTCAGGAGGAGCAATG	Human β-actin reverse

a. Introduced restriction sites are underlined. Amino acid spacer (GGGS) at the N-terminus of GFP is in bold.

### Cell culture, transfection and stable cell line generation

Normal mouse mammary gland cell line (NMuMG), HeLa and HEK293T cell lines were obtained from ATCC. Cells were routinely maintained in Dulbecco's modified Eagles medium supplemented with 10% fetal bovine serum (Gibco) and 100 μg/ml normycin.

Cells were seeded in 6 or 12 well plates one day before transfection. The transfection was performed using PEI as described previously [33]. For co-transfection, 0.2 μg p-mRFP-centrin1 plasmid and 0.8 μg MMTV Gag-derived plasmids were used.

To generate stable HEK293T cell lines expressing full-length MMTV, the p-CMV-MMTV-puromycin plasmid was linearized with *Pvu*I and transfected into HEK293T cells. Individual clones were selected with puromycin (Invitrogen).

### Cytoplasmic RNA preparation and mRNA quantification

HEK293T cells transfected with pUTR-Gag-GFP, pGag-GFP and pGag-GFP-CTE were collected 48 h post-transfection and the cytoplasmic fraction was prepared as described previously [34]. RNA was extracted using TRIzol LS reagent (Invitrogen) following the manufacturer's instructions. After DNA removal with DNase I digestion (Invitrogen), cDNA was synthesized from 500 ng of RNA by using random primers and SuperScript II reverse transcriptase (Invitrogen). The cytoplasmic Gag-GFP expression and relative quantification were performed with SYBR Green PCR Master Mix on a 7300 Real-Time PCR System (Applied Biosystems) using GFP primer pair 19/20 and human β-actin primer pair 21/22 as the housekeeping gene (Table 1), as described previously [35].

### Western blot analysis

Cell lysates were prepared from transfected and stable cells using RIPA buffer with complete proteinase inhibitor (Roche). About 5x10^6^ cells were collected and washed twice with ice-cold PBS. The cells were incubated with RIPA buffer on ice for 30 min and centrifuged at 20,000 x g for 30 min. Proteins in the supernatant were quantified using the BCA assay (Bio-Rad) and 30 μg of total protein were resolved by sodium dodecyl sulfate (SDS)-polyacrylamide gel electrophoresis (PAGE) and transferred to nitrocellulose membrane as previously described [36]. Immuno-detection was performed using the primary antibodies: rabbit anti-GFP (obtained from Dr. Luc G. Berthiaume, University of Alberta, Canada), rabbit anti-actin (Abcam), mouse anti-MMTV CA monoclonal primary antibodies (kindly provided by Dr. T. Golovkina, University of Chicago) and IRDye secondary antibodies: goat anti-mouse and goat anti-rabbit (LI-COR). Reacting membranes were visualized with a LI-COR Odyssey infrared imaging system.

### Cell immunostaining and fluorescent microscopy

For immunostaining, cells growing on coverslips were fixed 48h post-transfection in 2% paraformaldehyde for 5 min at room temperature and with ice-cold methanol at -20°C for 10 min. Fixed cells were then permeabilized with 0.1% Triton X-100 in PBS. MMTV Gag and γ-tubulin were detected with the mouse monoclonal anti-MMTV CA antibody (1:200 dilution) and a rabbit polyclonal anti-γ-tubulin antibody (1:400 dilution, Sigma-Aldrich), respectively. For fluorescent secondary antibody staining, a fluorescein (FITC) and Texas Red-conjugated anti-mouse and anti-rabbit immunoglobulin G (1:400 dilution) were used, respectively. Cell nuclei were stained with DAPI. The coverslips were then mounted in FluorSave Reagent (Calbiochem) prior to imaging. For fluorescent cell imaging, cells grown on 18 mm cover slides were incubated with Hoechst (1:200 dilution) for nuclear staining half hour before fixing with 2% paraformaldehyde and washed. The cover slips were mounted in FluorSave Reagent (Calbiochem) prior to imaging. All images were obtained with a Leica SP5 confocal laser-scanning microscope with sequential scanning settings. Images acquired were analyzed with Leica confocal software and Adobe Photoshop CC.

### TEM analysis

For TEM, stable MMTV and Gag-GFP transfected HEK293T cells were fixed in plates with modified Karnovsky fixative, 48h post-transfection (2% paraformaldehyde / 2% glutaraldehyde in 0.1M Na cacodylate buffer) for 1h. Cells were then scrapped and pelleted and post-fixed in 1% osmium tetroxide for 1h on ice in a dark fume hood. *En block* was stained in 1% uranyl acetate by rocking in the dark at room temperature for 1h. Dehydration through a series of graded ethyl alcohols (70 to 100%) was followed by 2x 10–15 min incubation at room temperature with propylene oxide, and overnight in 50/50 mixture of propylene oxide and embedding media (Embed 812) under vacuum. Pellets were then transferred to pure embedding media under vacuum for 6–8 h and placed into the beam and incubated at 60°C for 48 h. 70 nm sections were prepared using a Reichert-Jung Ultracut E Ultramicrotome. A Philips 410 equipped with a MegaView camera was used for imaging.

## Results

### The MMTV 5’ UTR enhances Gag-GFP accumulation

Subcellular localization studies of MMTV Gag have been hampered by insufficient expression levels from Gag-encoding constructs, which is due in part to the lack of *cis*- and *trans*-regulatory stabilizing elements that facilitate export of unspliced mRNA from the nucleus to the cytoplasm [37, 38]. In contrast, the expression of MMTV Gag has been enhanced with the insertion of the cytoplasmic transport element (CTE) sequence of the M-MPV [38]. The 5’ UTR upstream of the MMTV Gag ORF contains a regulatory element similar to an internal ribosomal entry site [39] and we hypothesized that insertion of the 5’ UTR upstream of the Gag ORF could increase protein expression and/or stability.

**Fig 1:**
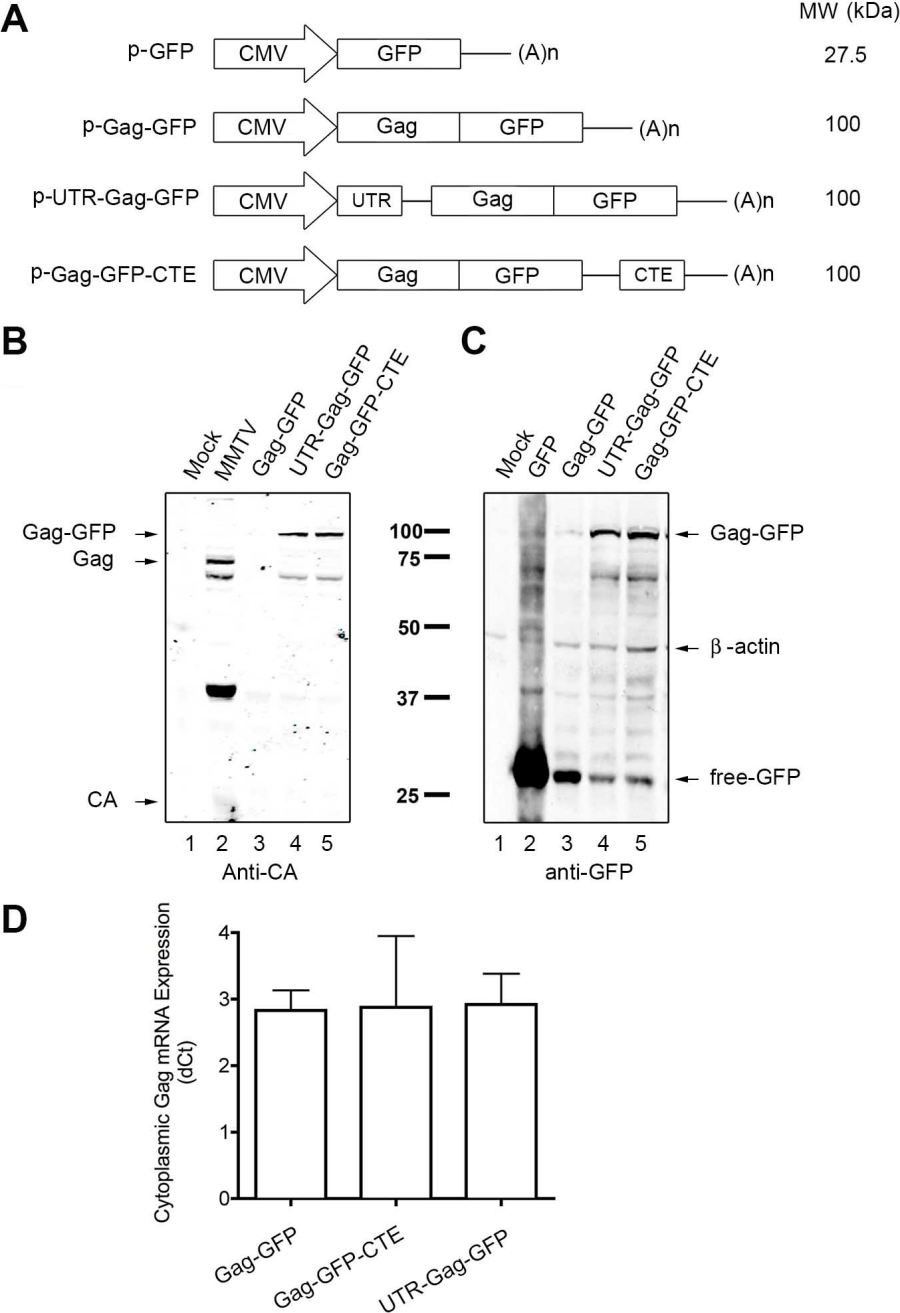
MMTV Gag-GFP expression is enhanced by the insertion of the 5’UTR of MMTV or CTE of M-PMV cis-regulatory elements. (**A**) Schematic representation of the expression vectors p-EGFP, p-Gag-GFP, p-UTR-Gag-GFP and p-Gag-GFP-CTE. The approximate size of each predicted protein is shown. All vectors contained the human cytomegalovirus immediate early promoter (CMV) and a polyadenylation signal (A)n. (**B**, **C**) HEK293T cells were transfected with mock (lane 1), MMTV (lane 2), Gag-GFP (lane 3), UTR-Gag-GFP (lane 4) and Gag-GFP-CTE (lane 5) plasmids two days prior to lysis. Proteins were resolved on SDS-PAGE and detected through western blot using anti-MMTV CA (anti-CA) or anti-GFP antibodies, using anti-β-actin as a loading control. Black arrows point to the bands corresponding to expected proteins (names included beside arrows). (**D**) HEK293T cells were transfected with the respective plasmids two days prior to cytoplasmic RNA extraction. Gag-GFP mRNA was quantified with RT-qPCR. Relative expression of the Gag-GFP mRNA to β-actin mRNA (ΔCt) in the cytoplasmic fraction is shown on the *y-*axis (n = 3).

We constructed expression vectors with the Gag ORF fused to GFP under the control of the cytomegalovirus immediate early promoter (Gag-GFP; Fig 1A). The MMTV 5’ UTR was then inserted upstream of the recombinant ORF (UTR-Gag-GFP) and we also placed a cassette containing the M-MPV CTE at the 3’ end of the recombinant ORF (UTR-Gag-GFP-CTE) [38]. Then HEK293T cells were transfected with plasmids containing Gag-GFP with or without the enhancer elements as well as a plasmid encoding the MMTV proviral genome. The protein expression was assessed by western blot using either an anti-MMTV CA antibody or an anti-GFP antibody two days post-transfection. While the Gag-GFP protein was undetectable in lysates from cells transfected with Gag-GFP (Fig 1B), the insertion of MMTV Gag 5’ UTR dramatically increased the accumulation of Gag-GFP to levels similar to those with M-PMV CTE (Fig 1B and 1C). Free GFP was also found with all plasmids encoding Gag-GFP, but this was not investigated further since most of the signal corresponded to Gag-GFP in the case of UTR-Gag-GFP or Gag-GFP-CTE.

Quantitative real time RT-PCR did not show significant differences in the level of mRNA from UTR-Gag-GFP, Gag-GFP-CTE or Gag-GFP (Fig 1D), suggesting that enhancement in Gag-GFP accumulation occurred at the translational or post-translational level.

### Gag-GFP localizes to pericentriolar regions

**Fig 2:**
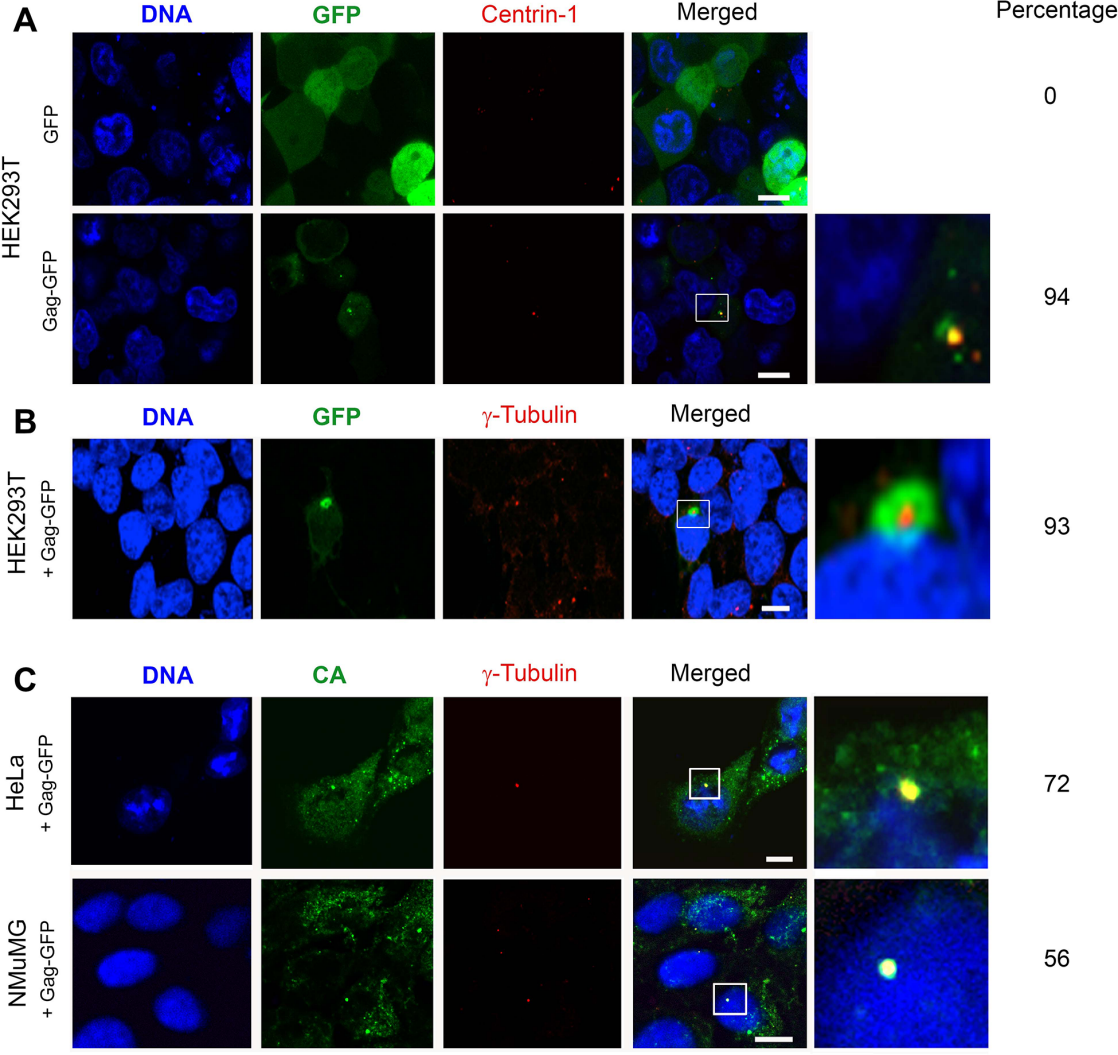
A 3’ fusion MMTV Gag-GFP localizes to the pericentriolar region. (**A**) HEK293T cells were co-transfected with a plasmid encoding the centriolar marker centrin-1 fused to mRFP (pmRFP-centrin1) and plasmids encoding free GFP (upper panel) or p-UTR-Gag-GFP (lower panel). Free GFP accumulated primarily in the nucleus and more diffusely in the cytoplasm, while Gag-GFP signal was observed within the cytoplasm with a substantial fraction co-localizing with centrin-1. (**B**) The same pattern was observed following p-UTR-Gag-GFP transfection with centrioles labeled with anti-γ-tubulin antibody. (**C**) The typical cytoplasmic distribution of scattered CA protein was observed in HeLa and NMuMG cells transfected with p-UTR-Gag-GFP, as reported by others. A fraction of the Gag protein also colocalized with centrioles labeled with anti-γ-tubulin antibodies. Right-most column represent a close-up view of the region framed in white in the “Merged” column. Nuclei were stained with DAPI [Bar = 10um]. The percentage of cells exhibiting pericentriolar localization of Gag is indicated on the right (n = 100).

Since the CTRS sequences of M-MPV and FV have been shown to direct pericentriolar localization of their cognate Gag proteins [12–16], we investigated whether the same process was relevant with MMTV Gag assembly in multiple cell types. Centrin-1 and γ-tubulin were used as centriolar markers as both proteins are specifically located at the centrosomes [40, 41]. In HEK293T cells, fluorescence from a free GFP expression vector concentrated in nuclei and more diffusely in the cytoplasm (Fig 2A a; upper panel). Gag-GFP was observed within the cytoplasm with perinuclear foci that co-localized with the centriole marker mRFP-centrin-1 (Fig 2A; lower panel) or with anti-γ-tubulin labeled centrioles (Fig 2B). Similarly, in HeLa and NMuMG cells (Fig 2C) punctate signal detected with the anti-CA antibody was observed in a cytoplasmic distribution as reported by others [26, 42]. A bright signal co-localizing with γ-tubulin was observed in both cell types indicative of pericentriolar localization.

**Fig 3:**
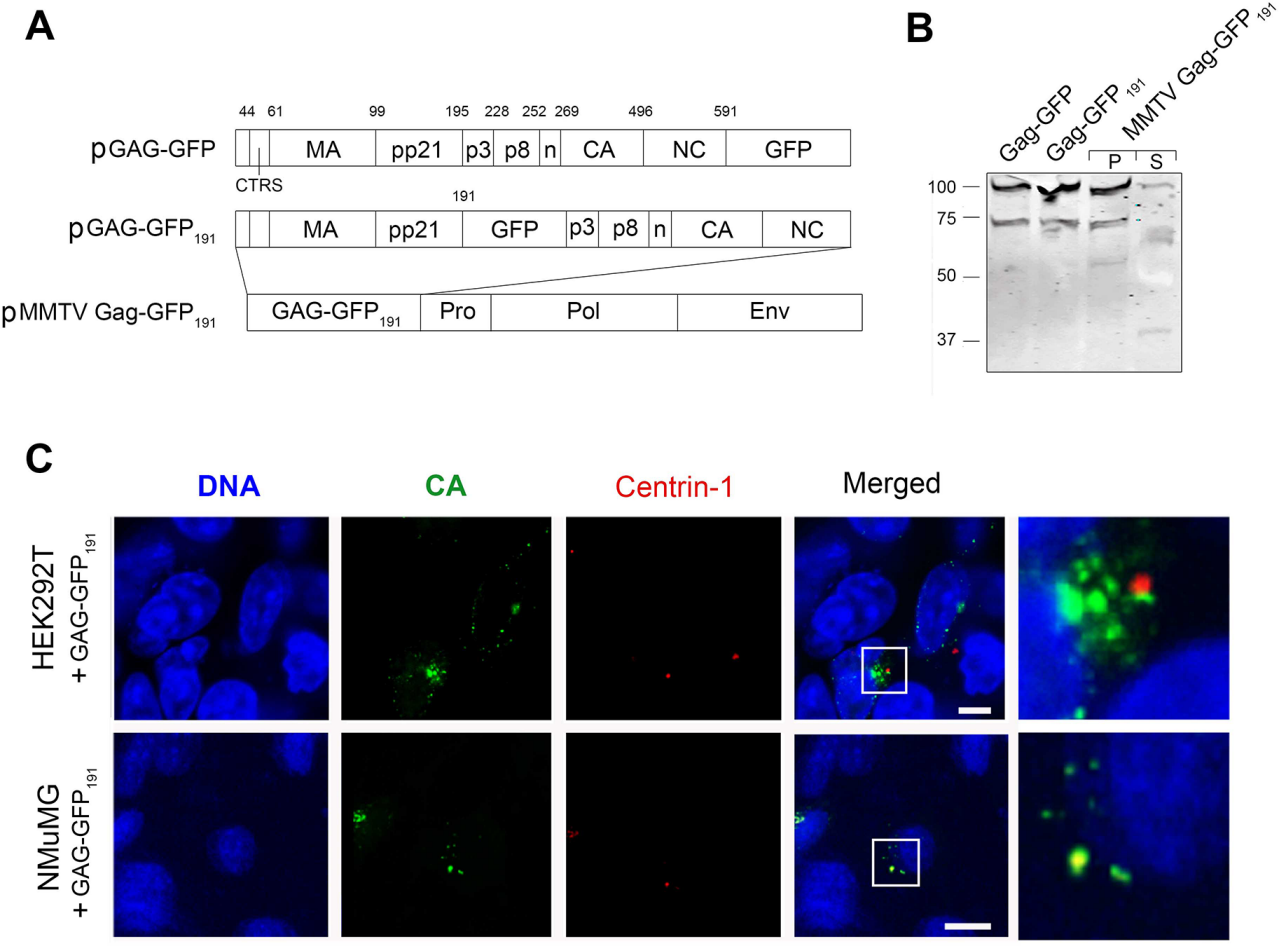
MMTV Gag-GFP expressed as full-length genome also localizes to the pericentriolar region. **(A)** Schematic representation of the Gag-GFP, Gag-GFP_191_ and MMTV Gag-GFP_191_ with CTRS and cleavage sites of Gag shown on top. (**B**) Western blot detection of Gag-GFP protein in HEK293T cells transfected with the Gag-GFP_191_ and MMTV Gag-GFP_191_ using anti-GFP antibodies [Gag-GFP protein in the pellet (P) and supernatant (S) indicative of virus particle production]. (**C**) Confocal images of HEK293T and NMuMG transfected with p-Gag-GFP_191_ and pmRFP-centrin1 obtained two days past transfection. Right-most column represent a close-up view of the region framed in white in the “Merged” column. DNA was stained with Hoechst [Bar = 10um].

We did not find viral-like particles that may form in the absence of virus nucleic acids, in the Gag-GFP transfected 293T cells by EM. Therefore, it is possible that the pericentriolar signals were protein aggregates, which are targeted to the pericentriolar region through the aggresome pathway. Thus, we generated an additional GFP fusion construct, Gag-GFP_191_, in which the GFP domain was inserted between the pp21 and p3 ORFs (Fig 3A). This protein was expressed at similar levels to that of other Gag-GFP constructs. Furthermore, the new fusion was functional in MMTV assembly as we could detect this fusion protein in the supernatant of cells transfected with the MMTV clone containing the Gag-GFP_191_ cassette (MMTV Gag-GFP_191_) (Fig 3B). We also detected the pericentriolar localization of the Gag-GFP_191_ construct in both HEK293T and NMuMG cells (Fig 3C), suggesting pericentriolar targeting is an intrinsic property of the MMTV Gag protein.

### The CTRS domain targets MMTV Gag to the pericentriolar region

**Fig 4:**
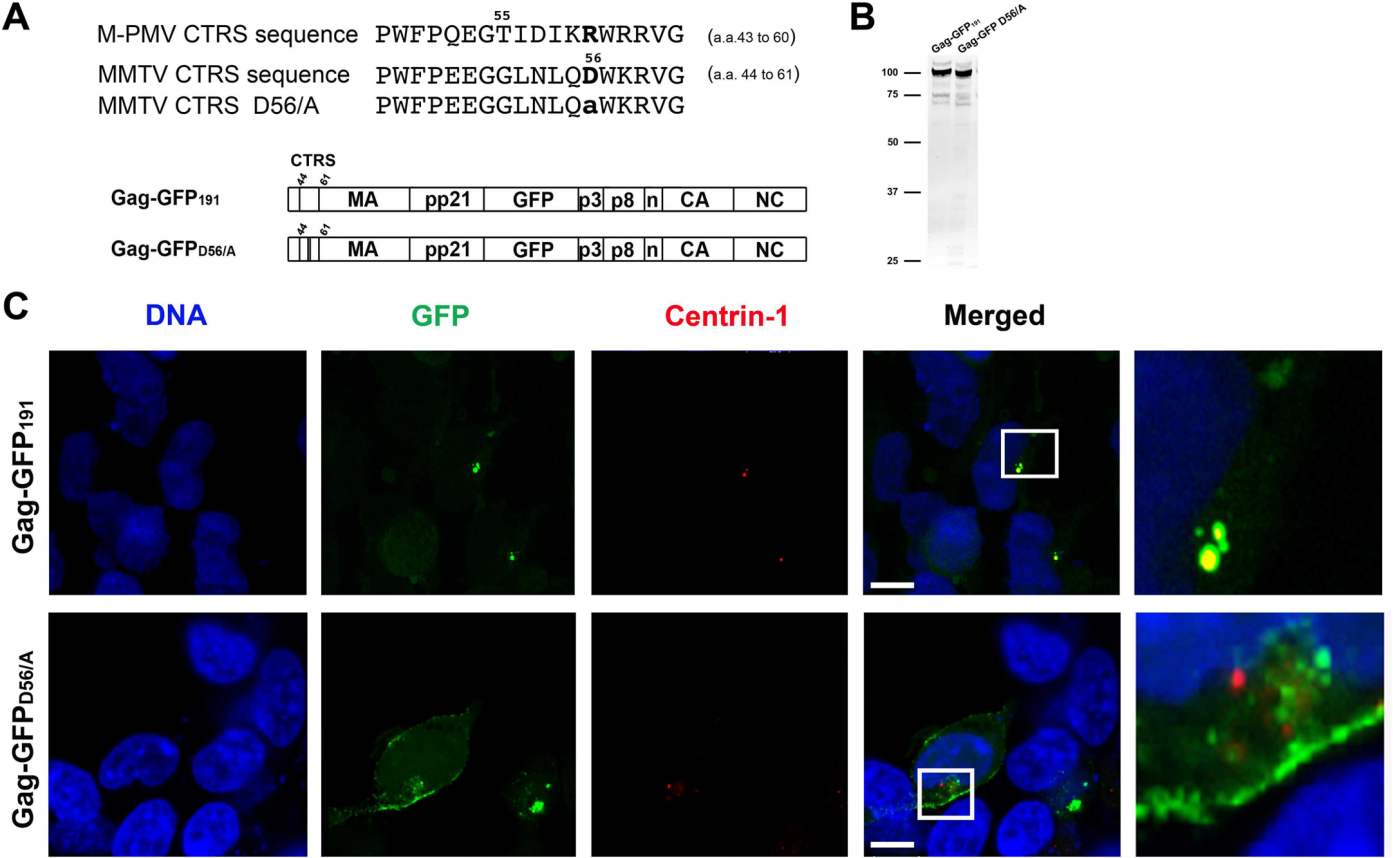
The CTRS domain within the MA is sufficient for pericentriolar localization of MMTV Gag. **(A)** The peptide sequence of the CTRS of MMTV and M-PMV are shown on the top above a schematic representation of the Gag-GFP_191_ and the Gag-GFP_D56/A_ mutant. **(B)** HEK293T cells were transfected with plasmids encoding Gag-GFP_D56/A_ or Gag-GFP_191_ two days prior to lysis and then resolved on SDS-PAGE to demonstrate protein production by Western blot analysis using an anti-GFP antibody. **(C)** HEK293T cells were co-transfected with pmRFP-centrin1 and the Gag-GFP_191_ or Gag-GFP_D56/A_ encoding plasmid. The intact CTRS domain was sufficient to localize a proportion of Gag protein proximal to the pericentriolar region, while the D56/A mutant disrupted targeting and the Gag-GFP was observed at the plasma membrane. Right-hand panel shows a magnified view of the region framed in white from the “Merged” panel. DNA was stained with Hoechst [Bar = 10um].

The MMTV MA includes a stretch of 18 amino acids resembling the M-PMV CTRS (Fig 4A). This retention signal is important for M-PMV assembly as a single amino acid substitution at the position 55 (R55/W) redirects capsids from the cytoplasm to the plasma membrane. To test whether the MMTV CTRS has analogous function, the Gag-GFP_D56/A_ fusion construct was generated and assessed in HEK293T cells by Western blot using anti-GFP antibodies (Fig 4A and 4B). The Gag-GFP_191_ and Gag-GFP_D56/A_ plasmids were separately co-expressed with the mRFP-centrin1 plasmid in HEK293T cells. Whereas Gag-GFP_191_ was localized to the pericentriolar region, the GFP signal of Gag-GFP_D56/A_ was redirected to the plasma membrane, indicating that the CTRS domain was sufficient for pericentriolar targeting (Fig 4C).

### MMTV Gag localizes to the pericentriolar region during virus infection

**Fig 5:**
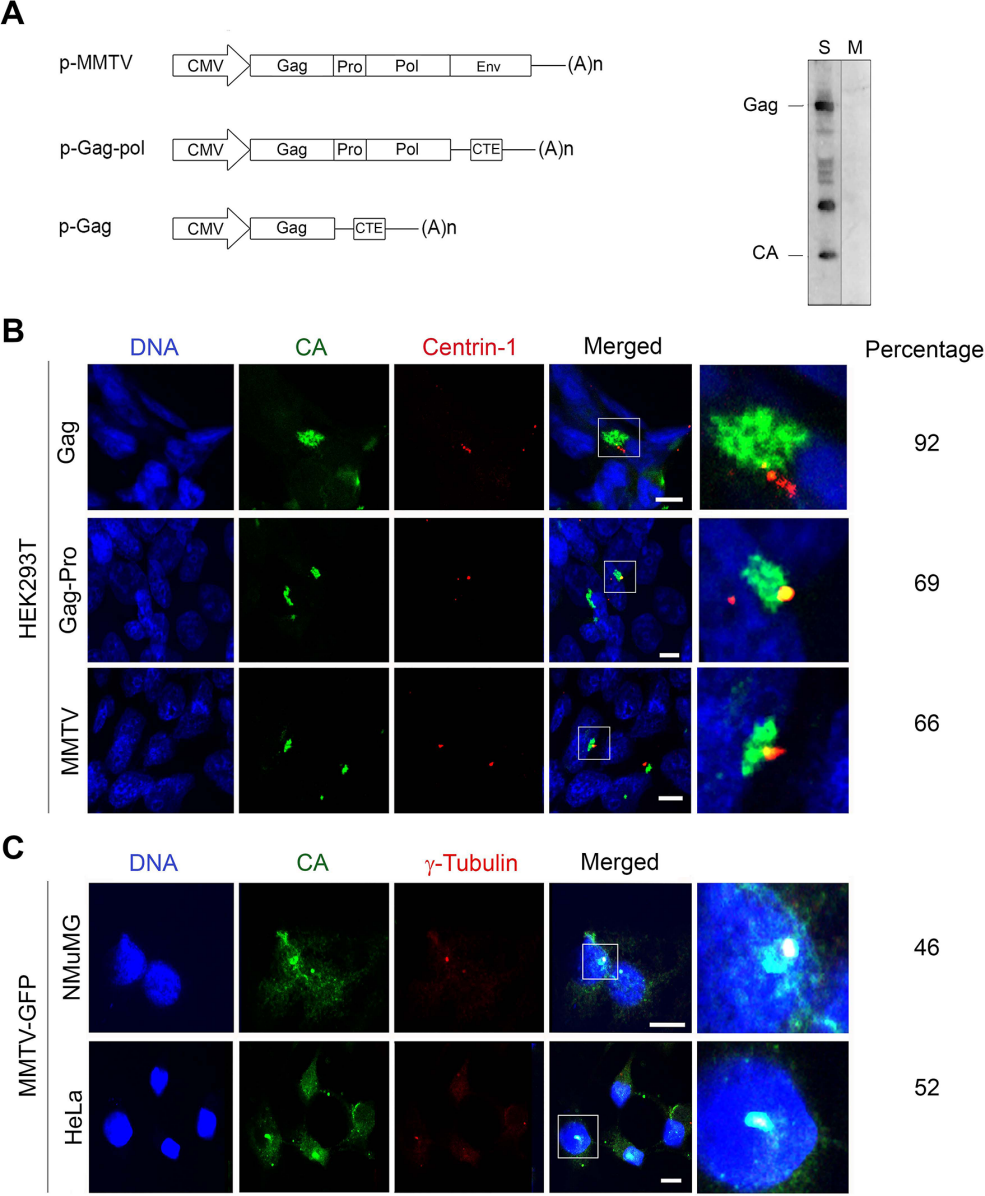
MMTV Gag localizes to the pericentriolar region in cells infected with MMTV. (**A**) Schematic representation of vectors used in this experiment, which encode Gag, Gag-Pro-Pol or the full-length MMTV genome. Western blot detection of the MMTV capsid (CA) domain in the Gag protein were observed in the supernatant (S) from HEK293T cells transfected with the full-length MMTV clone, whereas supernatants from mock (M) untransfected HEK293T showed no reactivity. (**B**) HEK293T cells were co-transfected with p-mRFP-centrin1 (Centrin-1) and either p-Gag, p-Gag-Pro or p-MMTV plasmids two days prior to fixation and staining with anti- MMTV CA antibodies (CA). Localization of MMTV Gag in the pericentriolar region was observed with all three viral plasmids. (**C**) NMuMG and HeLa cells persistently infected with MMTV encoding GFP in the 3’ UTR (MMTV-GFP ref [43]) also showed localization of the Gag protein around centrioles. In **B** and **C**, the panels on the right represent a close-up view of the region framed in white in the “Merged” panels. The percentage of cells exhibiting pericentriolar localization of Gag is indicated on the right (n = 50–100). DNA was stained with Hoechst [Bar = 10um].

We then investigated whether transfection of plasmids encoding either MMTV Gag alone, Gag-Pro-Pol or full-length genome had the same potential to localize to the pericentriolar region observed with Gag-GFP. We confirmed that transfection with full-length genome resulted in viral particle assembly and production by the detection of MMTV CA proteins in supernatant fractions (Fig 5A). We then co-transfected HEK293T cells with the plasmids encoding Gag, Gag-Pro-Pol or MMTV and the mRFP-centrin-1 plasmid and observed pericentriolar localization as previously seen with Gag-GFP (Fig 5B). Pericentriolar localization of MMTV Gag was also observed in HeLa and NMuMG cells (Fig 5C) chronically infected with MMTV-GFP (a replication competent MMTV virus encoding GFP in the 3’ UTR ref [43]). Together, these experiments demonstrate that Gag pericentriolar localization is a *bona fide* event during the virus life cycle.

**Fig 6:**
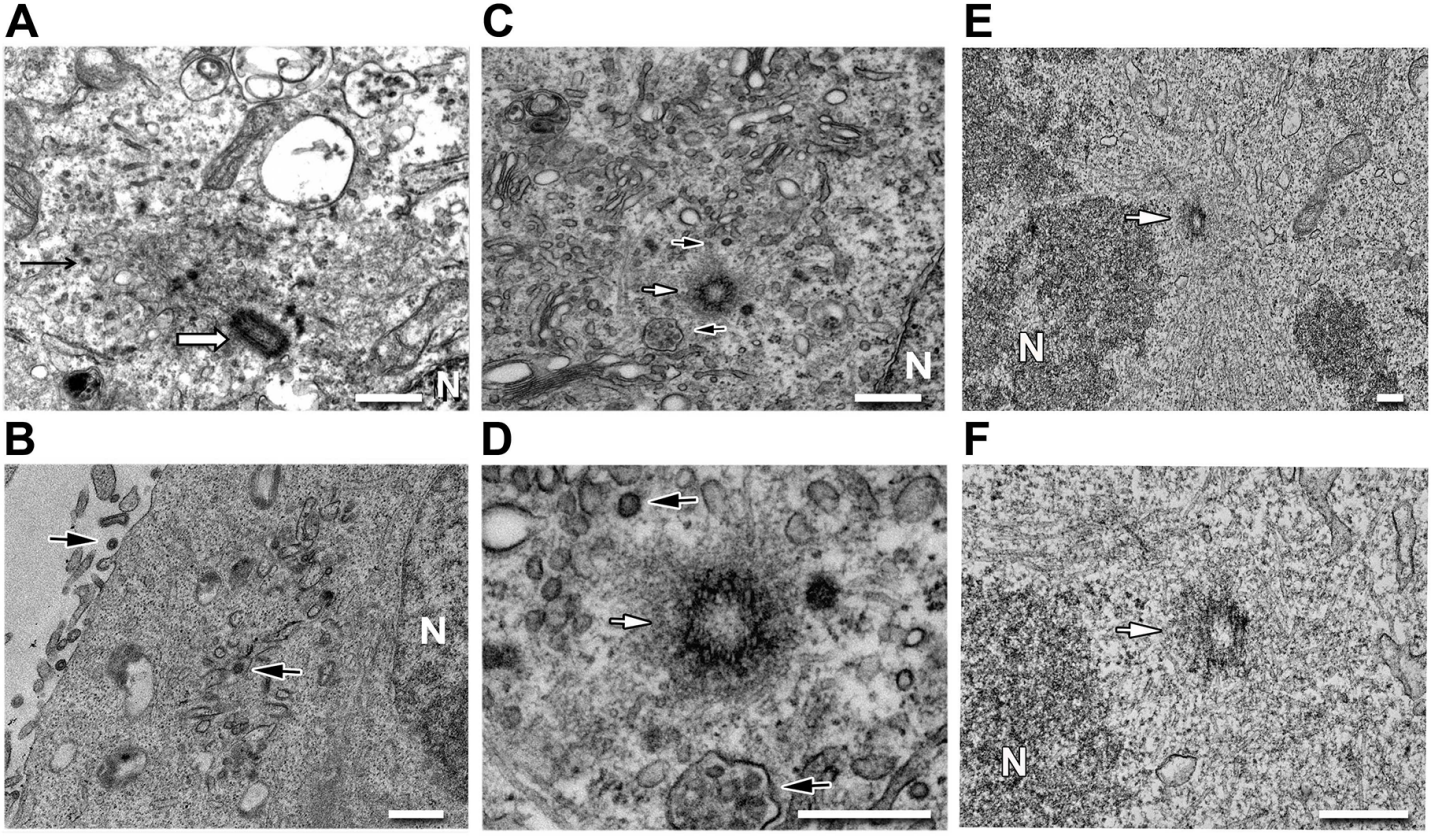
Immature MMTV capsids were found to accumulate at the pericentriolar region of HEK293T cells under TEM. (**A**) Immature capsids (black arrow) accumulated adjacent to the pericentriolar region (white arrow) in cells expressing Gag-GFP_191_. (**B**) Budding viruses at the plasma membrane and intracellular immature capsids (black arrows) were observed in cells expressing the full-length MMTV (p-MMTV in Fig 5). (**C**) Immature capsids and membrane vesicles containing virus like particles (black arrows) were observed in the same cells as **B** in close proximity to the centriole (white arrow). (**D**) Magnified view of the pericentriolar region of **C**. (**E**) No virus-like particles were observed in the pericentriolar region in cells transfected with the pEGFP plasmid. (**F**) Magnified view of the pericentriolar region of **E**. [N represents the nucleus. Bar = 500nm].

In transmission electron microscopy experiments we observed viral-like particles in the pericentriolar region of HEK293T cells expressing Gag-GFP_191_ (Fig 6A) or full length MMTV (Fig 6B, 6C and 6D). In contrast, we did not detect virus-like particles in cells expressing EGFP alone (Fig 6E and 6F). We also observed virus particles budding at the plasma membrane of HEK293T cells stably expressing MMTV along with the presence of extracellular viruses (Fig 6B). In sections with discernable centrioles, immature capsids were found to have assembled in close proximity (Fig 6A, 6C and 6D). Immature capsids-like structures were mostly confined to areas surrounding membranous tubules and to vacuole-like structures. Collectively, our results show that MMTV Gag exhibits a punctated subcellular distribution, but a substantial fraction localizes to the vicinity of centrioles, as reported for other retroviruses [12–16].

## Discussion

Herein, we show that transient expression of the MMTV Gag protein fused to a fluorophore (Gag-GFP) requires the presence of its cognate 5’ UTR. This is in agreement with the inability of the endogenous murine provirus, Mtv1 or the human derived HBRV Gag proteins to stably accumulate when expressed without enhancers [37]. Indeed, translation of Mtv1 or HBRV Gag requires nuclear events that involve Rem and Rem-responsive elements. We managed to express our Gag-GFP recombinant protein in the absence of Rem, but it required the presence of the MMTV 5’ UTR. Thus, it is likely that the MMTV 5’ UTR contains post-transcriptional regulatory elements that recruit nuclear factors needed to overcome translational inhibition of the MMTV gag mRNA, as reported for other retroviruses [44]. The enhanced translation of the Gag-GFP mRNA could also be due to the internal ribosomal entry site (IRES) recently identified in the 5’ UTR of MMTV [39]. In agreement with this observation, we found that the enhancement of Gag accumulation is not due to increased levels of gag-GFP mRNA in the cytoplasm, which is contrary to what has been reported for the MMTV envelope protein [45]. While further studies will be required to unravel the underpinning molecular mechanisms of ectopic Gag expression, we focused on the subcellular localization of the MMTV Gag protein in this study.

We provided several lines of evidence supporting the notion that the MMTV Gag is targeted to the pericentriolar region, which we believe to be a *bona fide* event during MMTV infection. First, recombinant proteins containing the full-length Gag fused to GFP accumulated at the pericentriolar region. Second, either the Gag protein alone or Gag expressed from *gag-pro-pol* or the full length MMTV genome co-localized with centriolar markers. Third, consistent with fluorescence microscopy analyses, TEM images confirmed that MMTV immature capsids accumulated at the pericentriolar region. The detection of MMTV virus like particles adjacent to the centriolar region by TEM with pGag-GFP_191_ and p-MMTV is an important finding because it is possible that tagging the Gag protein with GFP may alter Gag assembly due to the size of the GFP protein. In agreement, we observed no differences in Gag localization using co-transfection of GFP tagged and untagged Gag constructs (data not shown).

Pericentriolar accumulation and assembly of retroviral Gag proteins has been described for M-PMV, HFV and JSRV [13, 14, 46, 47], where the targeting for M-PMV and FV capsids is mediated by the CTRS signal present in their respective MA domains. For both viruses, a single amino acid substitution (R55/W) in the CTRS changed the localization of the Gag protein [13, 14]. Similarly, Gag-GFP_D56/A_ mutation in the MMTV CTRS relocated the MMTV Gag to the plasma membrane, suggesting a similar mechanism for pericentriolar targeting between viruses.

The pericentriolar accumulation of the MMTV Gag protein observed in this study is comparable with observations on M-PMV and JSRV Gag protein assembly [15, 47, 48]. However, we also observed a fraction of the Gag-GFP protein forming diffuse punctate signal in the cytoplasm, occasionally juxtaposed to the nuclear membrane. The latter is in agreement with Bann and colleagues [26], who found MMTV Gag in association with the YB-1 protein at P bodies and stress granules. Interestingly, a recent publication by Kawaguchi et al. reports that a phosphorylated isoform of YB-1 predominantly accumulates at the centrosome region during metaphase and this process is integral for centrosome maturation with the assembly of the microtubule array [27, 49]. Together, the data suggest that the MMTV Gag protein may be drawn toward the pericentriolar region by the combined interaction of phosphorylated YB-1 and the localizing effects of the CTRS. However, the exact biological relevance of this hypothesis remains to be resolved.
